# Longitudinal trajectories of mental health problems and their association with reoffending in a Dutch pre-trial prison cohort

**DOI:** 10.3389/fpsyt.2022.976832

**Published:** 2022-09-09

**Authors:** Michael Weber, Stéphanie Baggio, Leonel C. Gonçalves, Paul Nieuwbeerta, Anja J. E. Dirkzwager

**Affiliations:** ^1^Department of Psychology, University of Konstanz, Konstanz, Germany; ^2^Canton of Zurich, Department of Justice and Home Affairs, Office of Corrections and Rehabilitation, Zurich, Switzerland; ^3^Division of Prison Health, Geneva University Hospitals, University of Geneva, Geneva, Switzerland; ^4^Institute of Primary Health Care (BIHAM), University of Bern, Bern, Switzerland; ^5^Institute of Criminal Law and Criminology, Leiden University, Leiden, Netherlands; ^6^Netherlands Institute for the Study of Crime and Law Enforcement, Amsterdam, Netherlands

**Keywords:** mental health, distress, prospective cohort study, detained persons, recidivism, group-based trajectory modeling, changes

## Abstract

The burden of mental health problems in detained persons is high. At the same time, mental health problems are discussed as possible predictors of criminal recidivism. During detention, mental health tends to improve. The aims of the study were twofold: First, to identify group-based trajectories of mental health problems over the course of detention; second, to test the association between trajectories and criminal recidivism. A prospective cohort of 1,904 adult males detained in Dutch pre-trial detention facilities was assessed at three time points after imprisonment (week 3, month 3, and month 9). Mental health problems were measured using the Brief Symptom Inventory. Recidivism was defined as reconviction and re-incarceration up to 18 months post-release. We used group-based trajectory modeling and logistic regressions for the analyses. On average, self-reported mental health improved during incarceration. Two distinct groups of mental health trajectories were identified: The majority (81%) reported relatively low levels of mental health problems, remaining stable over time. A small group (19%) reported high distress after prison entry with improvements over time. Older age, pre-existing functional impairment due to alcohol or drug use, diagnosis of psychiatric disorders, debts, use of psychiatric care during detention, and a more severe experience of detention were associated with membership in the second group. Group membership did not predict reoffending. The study confirms prior findings illustrating a generally positive change in mental health symptoms during detention. The course of mental health was associated with pre-existing socio-demographic and psychological characteristics that seem worthy to be considered in correctional treatment plans. Changes in mental health did not result in better legal outcomes. An interesting avenue for future research would be to examine changes in specific mental health symptoms or disorders in relation to recidivism risk.

## Introduction

Compared to the general population, detained persons are more affected by mental health problems. This pattern has been shown robustly across different countries and subgroups of individuals in custody (1–6). At the same time, mental health problems are risk factors for criminal behavior (7, 8). Over the past decades, research on the prevalence and treatment of detained persons' mental health has gained increased attention. However, research on the longitudinal course of mental health problems during detention is scarce (9). In this study, we examine the course of mental health problems in a cohort of persons in pre-trial custody that was longitudinally analyzed, and the effect of different mental health trajectories on criminal recidivism. The results of our study can inform policymakers as well as prison staff about possible strategies aiming to improve detained persons' mental health with a special focus on reducing the risk of reoffending. In the following, we first summarize (1) the research on the relationship between mental health problems and recidivism, (2) the literature on the course of mental health problems during detention, and (3) recent findings on factors associated with changes in the course of mental health problems during detention.

### Mental health problems and criminal recidivism

In the literature, there is evidence of mental health problems as risk factors for criminal behavior including violent outcomes (7, 8). Various clinical conditions, including externalizing disorders like attention-deficit/hyperactivity disorder, disruptive behavior disorder, substance abuse, and personality disorder, as well as psychotic disorders, seem to be robust predictors for criminal recidivism (10–14). In a longitudinal study, support for a health-based model of desistance could be found, in which physical as well as mental health aspects lead to better work and family situations and finally to a reduced likelihood to recidivate (15). Furthermore, the Good Lives Model (GLM), a strength-based approach to offender rehabilitation (16) has been expanded to forensic mental health populations. Accordingly, mental health problems are the main obstacles to achieving primary goods like life, relatedness, knowledge, or excellence in work and play. Mental health problems are often opposed to a non-deviant life, as symptoms of mental illness can be considered a means to obtain primary goods, e.g., violent behavior in psychotic individuals can be seen as an attempt to save one's life, if he or she feels haunted by paranoid hallucinations. In mentally ill persons, however, primary goods are only reached partially, or, in the case of psychosis, within a strongly biased perception of reality (17, 18).

At the same time, there is evidence that adequate treatment developed to meet the psychiatric and criminogenic needs of offenders with mental health problems can reduce the risk of recidivism. Even among high-risk offenders, appropriate interventions can reduce the recidivism rate to a risk level reported in general offender populations (19–21).

### Course of mental health during detention

A typical course of mental health problems seems to be a higher burden at the time of imprisonment that tends to improve during detention (9, 22–25). Several reasons for the improvement of mental disorders in prison are discussed: Particularly detained persons suffering from psychotic disorders or substance use problems benefit from better access to physical as well as mental health care, restricted possibilities to use alcohol and drugs, and a clear structure given by the prison regime (25–28). In individuals with long-term sentences, changes like stabilization of depressive attitude, emotional instability, or decrease of hostility are discussed as possible factors leading to lower symptom load in the course of imprisonment (29).

### Predictors of mental health changes in detention

A number of factors can influence changes in the mental health status of detained persons. The method used to select relevant references as well as the flow diagram of resulting references can be found in Supplementary material 1 and Supplementary Figure 1.

#### Sociodemographic variables

It is a robust finding that older age, low level of education, and marginalized labor situation are associated with a number of adverse mental health outcomes in general populations (30, 31) as well as in populations of detained persons: Higher age is associated with a significant improvement in subjective wellbeing in individuals in pre-trial detention. In the same sample, subjective wellbeing significantly improved in individuals with housing problems and unemployment before imprisonment (22). Studying or working during imprisonment was associated with a strong improvement in the global severity index measured by SCL-90-R (24). Regarding the level of education, a higher burden of mental health problems in incarcerated youth is associated with a lower level of education (28).

Besides these standard demographic characteristics, debts are discussed as relevant factors for legal and mental health outcomes in detained individuals. Prisoners are affected by financial claims coming from different sources. Especially in the US, they include legal financial obligations like victim restitution, criminal fines and surcharges, court fees, incarceration charges, and fees for post-release supervision. The need for child support, the accumulation of preexisting debts that cannot be paid off while detained, as well as new debts incurred after release to cover living expenses represent common challenges that imprisoned individuals are faced with (32). Importantly, debts have a negative effect on financial wellbeing, employment, reentry, family structure, and mental health (32, 33). Furthermore, a strong association between debts and crime has been shown, whereby the two factors influence each other to a negative extent: debts have to be considered a risk factor for criminal recidivism, and crime is a risk factor for having debts (34).

#### Mental health

Looking at specific disorders, psychosis at prison entry was associated with slight symptom improvement (24) or remained stable (9). While the prevalence of mental health symptoms decreased among detained persons with depression, this was not observed among those with other mental illnesses (9, 25). Similarly, depression at baseline was associated with a strong improvement of the global severity index measured by SCL-90-R (24). At admission to pre-trial detention, subjective wellbeing significantly improved in individuals with a history of mental illness and alcohol and substance use disorders (35); In contrast, antisocial personality disorder has been shown to be a risk factor for a decrease in wellbeing during pre-trial detention (22).

Importantly, the initial level of subjectively perceived mental distress seems to affect the course of mental wellbeing during imprisonment: While detainees with especially high levels of mental health problems at beginning of imprisonment are reported to experience a significant decrease, those with initially low levels of mental distress have been shown to experience an increase (36). Furthermore, a high level of wellbeing was associated with features of aggression, dominance, and hostility (36). While better physical health during imprisonment as well as gains in physical health *after* release were associated with higher recidivism risk, better mental health *in* prison and especially an improvement in mental health *after* release were related to lower recidivism risk (37).

#### Correctional climate and subjective experience of detention

In a longitudinal study on young detained individuals, a negative perception of the correctional climate was the strongest covariate of mental health symptoms that showed incremental validity over that of personal variables (28). Furthermore, the deterrent effect of severe sanctions has been widely discussed (38, 39). However, recent findings indicate that a lower probability to reoffend may depend on procedural justice (40, 41). Sanctioning is associated with higher rates of offending when treatment was experienced as unjust, but not when treatment was experienced as just (42). Similarly, a positively perceived procedural justice predicted better self-reported mental health (43). Persons who felt treated in a procedural manner during imprisonment, also those who experience their imprisonment as more severe, were less likely to be reconvicted (44–46). However, other variables associated with perceptions of sanction severity, such as demographic characteristics, social involvement, and criminal lifestyle, were shown to explain the initially found negative effect of perceived severity on recidivism (46). Yasrebi-de Kom and colleagues (45) reported that a deterrent effect of sanction severity also depends on perceived procedural justice during detention: in a subgroup of first-time detained persons, the effect was only present if treatment by prison staff was judged as neutral to fair.

### Objective

There is a long research tradition on the association between mental health and criminal recidivism. In addition, changes in the mental health status of individuals while they are incarcerated have been empirically studied. However, there is no research line that brings both approaches together. That is, examining the influence of differential mental health trajectories on reoffending. The aims of the study were twofold. First, we aimed to identify group-based trajectories of mental health problems over the course of detention and the characteristics of the individuals pertaining to the different groups. Second, we sought to test the association between the trajectories identified in the first step with criminal recidivism measured at three different follow-up times. The findings of the present study may inform policymakers as well as prison staff about possible strategies aiming to improve detained persons' mental health with a particular focus on reducing the risk of reoffending.

## Materials and methods

### Sample

Data were used from the Prison Project, a nationwide, longitudinal, and prospective cohort study examining the development of criminal behavior and other life circumstances, including mental health, of males in pre-trial detention in the Netherlands. The sample consisted of adult males aged 18–65 years, who were born in the Netherlands, and who entered one of the 30 Dutch pre-trial detention centers between October 2010 and April 2011 and who were followed-up until April 2015.

### Study design

Demographic, personal, and psychological variables were assessed at three time points: 3 weeks after incarceration (T1), detained individuals were interviewed and asked to complete written questionnaires. Three months (T2) and 9 months (T3) after incarceration, they were again handed written questionnaires. Follow-up information on re-incarceration and re-conviction at three different time points after release (6, 12, and 18 months) were gathered from official national registration systems. Of the total target population, 71% (*n* = 2,837) could be approached and of these approached individuals, 67% agreed to participate in the interview, leading to a total response rate of 48% (*n* = 1,904). Of the interviewed persons, 92% also filled out the written questionnaire (*n* = 1,748). The sample size of persons providing information both in the interview and in the written questionnaire was *n* = 1,748 for T1, *n* = 838 for T2, and *n* = 265 for T3. At the 18-month follow-up, data for *n* = 1,641 individuals could be obtained. Detailed information on the sampling procedure and study design can be found in the description of the cohort profile (47). The authors followed the criteria of “The Strengthening the Reporting of Observational Studies in Epidemiology (STROBE) Statements: guidelines for reporting observational studies” (48) to make a complete and accurate report of the study.

### Measures

#### Study variables

##### Mental health distress

Mental Health problems were measured using the Dutch version of the Brief Symptom Inventory (BSI) (49, 50) total score. The BSI is a self-report instrument that has been shown to have good psychometric properties (51). It is composed of 53 items assessing the experience of mental health symptoms during the previous week (0 = not experienced at all, 4 = experienced a lot). The items are organized into nine subscales. The BSI has been used across different cultures and samples, including samples of detained persons (52, 53). The unidimensional factor structure has the most robust empirical support, meaning that the BSI measures a common latent construct. Interpretation of single sub-scales seems to be difficult (54–57). Therefore, we used the BSI total score as a measure of global psychiatric/psychological distress.

##### Predictors of mental health changes in detention

Based on the variables known to be associated with change in mental health status of persons while they are detained, the following variables were included as possible predictors: Age at start of detention; level of education (low = 0; medium/high = 1); labor situation before detention: currently working or studying (no = 0; yes = 1); having debts (0 = no; 1 = yes); type of index offense (non-violent = 0; violent = 1); functional impairment due to alcohol or drug use before detention: 4-item scale of alcohol and drug functioning (0 = all items coded negative; 1 = ≤ 1 item coded positive); any psychiatric diagnosis in the previous 12 months before detention (no = 0; yes = 1); psychiatric care while detained (no = 0; yes = 1); subjective experience of detention (mean of four items asking on a 5-point Likert-type scale to what extent participants agree or disagree with statements concerning their subjective experience of detention, e.g., “I feel safe in this institution”; 1 = strongly disagree; 5 = strongly agree; low values indicating poor, high values indicating good experience).

##### Criminal recidivism

Criminal recidivism was defined as re-incarceration 18 months after release from custody, as recorded in official national registration systems. For the purpose of sensitivity analysis, we examined also re-conviction, and we used varying follow-up periods of 6 and 12 months after release.

#### Statistical analysis

Group-based trajectory modeling (GBTM) is a kind of finite mixture modeling that identifies clusters of individuals following similar trajectories. GBTM assumes that there are distinct (unknown) developmental trajectories within the population, labeled as “groups.” It uses a multinomial strategy with maximum likelihood estimation to identify these groups. GBTM has been used in criminological research (58–60).

We used a censored normal distribution (Tobit model) with linear and quadratic trends and included time-stable covariates that could influence the probability of group membership. The model generates group-membership probabilities based on these covariates. Parameters defining the trajectories and group membership probabilities were estimated jointly. The number of groups was selected using the Akaike information criterion (AIC), Bayesian information criterion (BIC), likelihood (LL), entropy, and the number of participants in each class (> 5%). Missing values were handled with full-information maximum likelihood to use all available information for the estimation. We first described groups (shape and size) and factors associated with group membership to answer our first research question. We then tested whether group membership affected recidivism using logistic regression models to answer our second research question. Odd-ratios (OR) are reported. The significance level was set at α = 0.05.

## Results

### Descriptive statistics

Table 1 shows the descriptive statistics of study variables for the measurement points at week 3 (T1), month 3 (T2), and month 9 (T3). At the time of the first measurement, individuals were 30.4 years old (*SD* = 10.75). About one-third (36.5%; *n* = 631) had at least a medium education level. Slightly less than half of the participants (47.5%; *n* = 829) were engaged in work before imprisonment. The majority (57.0%; *n* = 1,079) had debts. Almost half of the individuals (45.9%; *n* = 802) had committed a violent offense before imprisonment. One-third (33.5%; *n* = 584) had substance use problems.

**Table 1:** Descriptive statistics at each time point.

	**T1**	**T2**	**T3**
Age^a^	30.37 (10.75)	-	-
Education (medium/high)^b^	36.5 (631)	-	-
Working or studying (yes)^b^	47.5 (829)	-	-
Debts (yes)^b^	57.0 (1,079)	-	-
Violent offending (yes)^b^	45.9 (802)	-	-
Previous functional impairment due to alcohol or drug use (yes)^b^	33.5 (584)	-	-
Any psychiatric disorder before detention (yes)^b^	38.1 (634)	-	-
Psychiatric care while detained (yes)^b^	23.8 (392)	54.3 (445)	49.6 (128)
BSI^a^	0.70 (0.71)	0.58 (0.54)	0.53 (0.60)
BSI^c^	0.47	0.42	0.32
Subjective experience of detention^a^	3.52 (0.94)	3.73 (1.05)	3.38 (1.11)

T1 = 3 weeks after incarceration, T2 = 3 months after incarceration, T3 = 9 months after incarceration;

a Means (standard deviations);

b Percentages (n);

c Medians.

More than one-third of individuals were diagnosed with any psychiatric disorder in the previous 12 months before detention (38.1%; *n* = 634). While less than one in four received psychiatric care in prison just after incarceration (23.8%; *n* = 392), about half were in psychiatric treatment at month 3 (54.3%; *n* = 445) and month 9 (49.6%; *n* = 128). The median BSI total score decreased from 0.47 shortly after incarceration to 0.42 in 3 months and 0.32 in 9 months after incarceration. The subjective experience of detention remained comparable over the three measurement points (see Table 1). The 18-month re-incarceration rate was 21.7%.

### Group-based trajectory modeling

#### Selecting the number of groups

GBTM allowed for a two- or three-group model. AIC, BIC, and LL were a little better for the three-group model (AIC: −2231.61 vs. −2191.23; BIC: −2277.52 vs. −2272.22; LL: −2214.63 vs. −2161.23), whereas the entropy was slightly higher for the two-group model (0.861 vs. 0.868). However, in the three-group model, there was a very small number of participants in one group (1.0%, *n* = 16), so we chose the two-group model.

**Figure 1:**
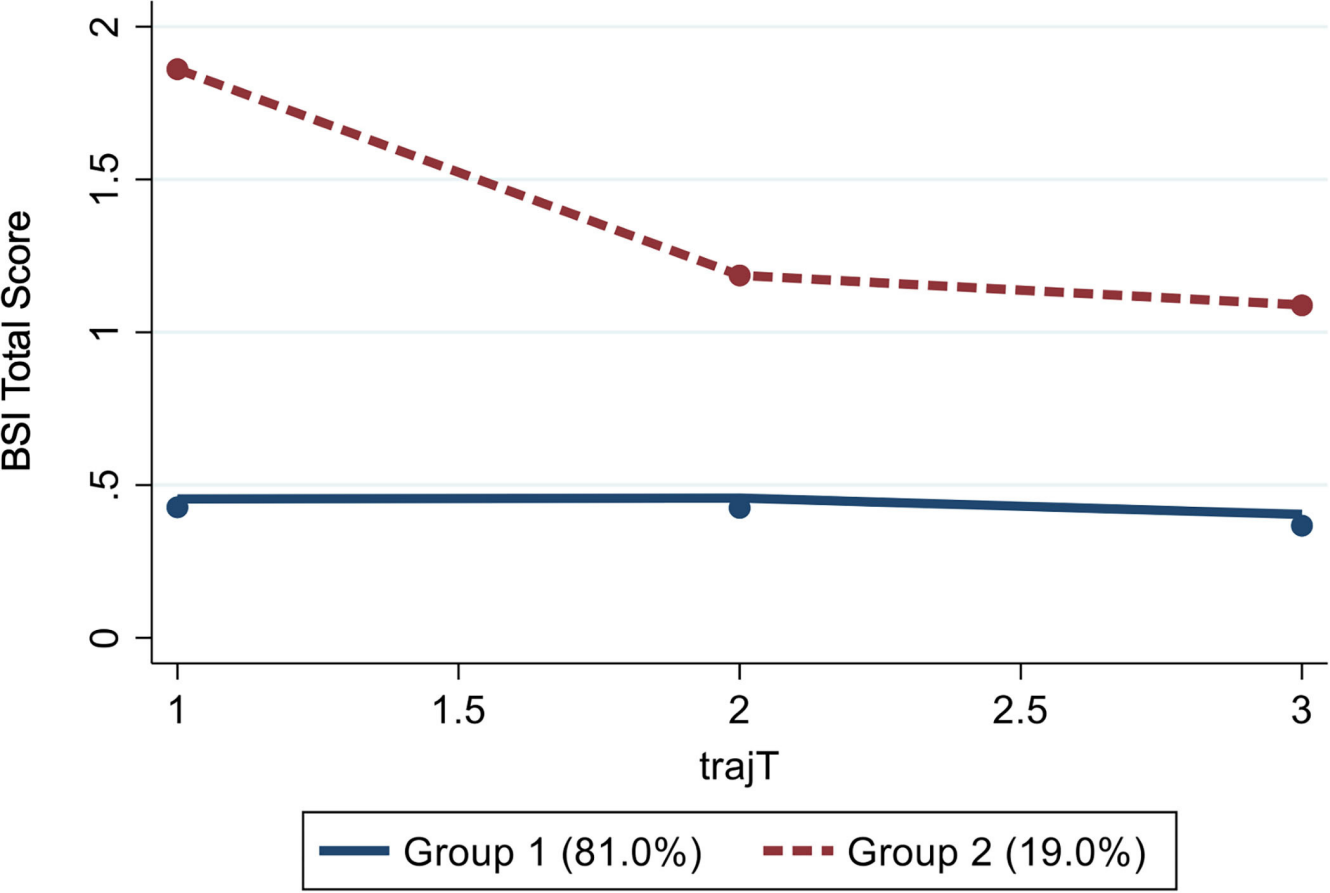
GBTM revealing two distinct groups of mental health trajectories (*N* = 1,635). BSI, Brief Symptom Inventory (Dutch version); trajT, trajectories over the three measurment points (T1, T2, and T3).

Based on these results, we identified one group with a low level of mental health problems stable over time including 81.0% of the sample, and one smaller group (19.0%) with a high initial level of mental health problems improving over time (significant linear and quadratic trends: *p* < 0.001; see Figure 1). The improvement already occurred between the first and second measurement point, i.e., within week 3 and month 3, and remained stable to the third measurement. The detailed model parameters are presented in Supplementary Table 1.

#### Predictors of group membership

Participants in group 2 were more likely to have psychiatric disorders diagnosed before detention (*p* < 0.001), previous functional impairment due to alcohol or drug use functioning (*p* < 0.001), and debts (*p* = 0.004). They were more likely to access psychiatric care during detention (*p* = 0.001) and to have a more difficult experience of detention (*p* < 0.001). In addition, participants in group 2 were older than participants in group 1 (*p* = 0.004). There were no significant differences in the level of education, labor situation, and being incarcerated because of violent offending. Detailed parameter estimates are presented in Table 2.

**Table 2:** Predictors for membership in trajectory group 2 (main analysis; *N* = 1,635).

**Variable**	**Coefficient**	**Standard**	***T***	***p***
		**error**		
Age	0.02	0.01	2.89	<0.001
Education (medium/high)	−0.12	0.18	−0.70	0.486
Work (yes)	−0.34	0.18	−1.87	0.061
Debts (yes)	0.52	0.18	2.85	0.004
Violent offending (yes)	0.10	0.17	0.61	0.544
T1 Substance use problems (yes)	0.88	0.18	4.85	<0.001
T1 Any psychiatric disorder (yes)	1.60	0.19	8.60	<0.001
Psychiatric care in detention (yes)	0.58	0.17	3.34	0.001
T1 Subjective experience of detention	0.65	0.10	6.52	<0.001

### Prediction of criminal recidivism

Logistic regression was run with group membership as a predictor variable. Results indicated that group membership does not predict re-incarceration within 18 months after release: χ^2^ (1, *N* = 1,640) = 0.54, *p* = 0.464; OR = 1.12, 95% CI = [0.82; 1.51], *p* = 0.461.

### Sensitivity analyses

As predictors are important to define group trajectories, we calculated a model including predictors in our main GBTM analysis. Still, we calculated an additional model without predictors. This model also revealed two groups with slightly different proportions (85% in group 1 and 15% in group 2, respectively).

Since control variables were already included in the GBTM model as represented by the changes in group trajectories, the initial regression model to predict criminal recidivism was run with group membership as the only predictor. When running a regression model including the control variables, none of the variables proved significant neither. Results were neither significant for 6- and 12-months recidivism (6 months: *OR* = 1.19, *p* = 0.384; 12 months: *OR* = 1.25, *p* = 0.173), nor if re-conviction was used as outcome criterion.

Finally, we ran the same models (GBTM and logistic regression) including only those participants who completed all three interviews (*n* = 223 after exclusion of individuals with missing values in one or more of the predictor variables). Similar to the main analysis, a two-group model with comparable proportions (83.2% in group 1, 16.8% in group 2) was preferred against a three-group model. Regarding possible predictors for group membership, fewer covariates proved significant, possibly due to a lack of power. Members of group 2 were more likely to have a diagnosis of any psychiatric disorder before detention (*p* = 0.004), have functional impairment due to alcohol or drug use (*p* = 0.032), and have a more difficult subjective experience of detention (*p* = 0.001). Logistic regression revealed no association between group membership and criminal recidivism in the reduced sample. Detailed information on the results of sensitivity analyses of the reduced sample is presented in the Supplementary Tables 2, 3, and Supplementary Figure 2.

## Discussion

### Differential trajectories of mental health

In this study, we first identified group-based trajectories of mental health problems over the course of imprisonment. Second, we tested the association of these trajectories with criminal recidivism. Our results challenge existing findings stating a generally positive mental health change in individuals while they are detained. Rather, change seems to be driven by a distinct group comprised of about one-fifth of the assessed individuals (group 2). They reported very high distress at the start of detention with significant improvement over time, but still at a higher level compared to the other trajectory group. Several socio-demographic and psychological factors were associated with group membership: First, higher age increased the probability to belong to group 2. Thus, our results add to findings of the particularly high burden of somatic and mental healthcare needs in the group of elder persons in detention; a subject that will gain attention since the number and the portion of this special needs group is growing (6, 61, 62). Unsurprisingly, suffering from at least one psychiatric disorder and having functional impairment due to alcohol or drug use before detention both were associated with experiencing a high level of mental distress close to intake, but also a significant improvement during detention. The latter finding is in line with prior research revealing positive change in detained persons suffering from conditions like substance abuse problems and depression (22, 24, 25). Having debts is a further predictor of membership in the highly-distressed group. This finding is consistent with prior research stressing the negative impact of debts on mental health (32). By contrast, there was no impact on labor situation (albeit just short of the significance threshold), level of education, and being detained because of violent offending on membership in one of the identified groups. The formerly found association of elevated wellbeing and aggressive features (36) could therefore not be complemented by the present findings; maybe violent index offense is too dissimilar from violent features during imprisonment and therefore was not associated with belonging to group 2. It is worth mentioning that the other non-significant variables both belong to the domain of social involvement before imprisonment; a feature that is associated with worsened mental health outcomes during imprisonment (22, 28). Unfortunately, it was just possible to include social integration before, but not during detention in the model (24).

Considering the positive direction of change in group 2, our findings indicate that individuals in this initially extremely burdened group may have benefited from their time in detention. This is supported by the higher probability that individuals in group 2 undergo psychiatric treatment while in detention, which may have a positive effect on mental health, possibly provided by better access to physical and mental health care, restricted possibilities to use substances, and the structured daily routine (25–28). Interestingly, persons in group 2 showed improvement *despite* their higher probability to judge detention as being more difficult—a factor that has been associated with adverse mental health outcomes (28). Yet, the level of psychological distress in this group was still higher compared to the other group that reported relatively low distress remaining stable over time. Moreover, both groups were exposed to higher levels of psychiatric distress compared to the Dutch general population (49).

The present study underscores the importance of providing multidisciplinary care, especially for incarcerated individuals who suffer from serious mental health problems. Interventions should comprise medical, mental health, and substance use treatment, during and after incarceration; reducing existing treatment barriers and increasing recovery outcomes are recommended as the main focus of correctional treatment (63). (Psycho-)therapeutic interventions should be accompanied by research on their effectiveness to allow for specific treatment guidelines or recommendations on required human resources, as such evidence is currently scarce (64).

### Mental health changes and criminal recidivism

To our knowledge, our study is the first to examine the direct effect of changes in self-reported mental distress on the probability to reoffending. We found that those changes are not predictive of criminal recidivism. This main finding is valid for different outcome criteria (i.e., re-incarceration and re-conviction) and time spans (i.e., 6-, 12-, and 18 months post-release). Results may be interpreted in the way that psychiatric illness, or at least symptoms of specific disorders, are risk factors for criminal recidivism (8, 11, 12), but not short-term changes in overall mental distress. Existing research shed light on the role of different measurements of psychiatric illness, or symptoms of specific disorders when examining the course of mental health during imprisonment. Prior research could show that the BSI is sensitive to detecting global psychological distress, best to be interpreted as mild forms of psychopathological symptoms. Our results are compatible with prior findings that symptoms of specific mental disorders seem to be more affected by a positive change (e.g., depression and psychotic disorders (24, 25). On the contrary, symptoms of rather pervasive disorders like personality disorders as well as subjectively reported mental distress as measured by, e.g., the SCL-90-R and also the BSI seem more resistant to change (22, 65).

An effect of mental disorders manifested by symptoms that are highly apparent to the environment, including psychosis and externalizing disorders, on criminal recidivism has been robustly demonstrated (11–14, 66, 67). Therefore, it seems possible that changes in symptoms of severe disorders will affect the probability to reoffend much more than just the average of subjectively reported mental health problems. An interesting approach for future research would be to examine mental health changes in sentenced individuals, who seem to be more affected by a positive symptom change than those in pre-trial detention (25). Regarding the length of the observation period, the significant improvement in group 2 already occurred within a short time frame of slightly more than 2 months after the first measurement and 3 months after prison entry. This indicates that changes can occur relatively quickly and thus be measured in individuals detained in short-term pre-trial detention.

### Limitations

This study has several limitations. This applies first of all to the composition of the sample: Only males in pre-trial detention that were born in the Netherlands were included. Consequently, it remains unclear to what extent the findings can be generalized to females or to first-generation immigrants in detention.

Second, the findings may be valid only for the Dutch correctional system with its relatively liberal and decent prison conditions and comparably low prison population rate. Moreover, short-term confinements of <6 months account for the majority of prison sentences served in the Netherlands. Consequently, a considerable portion of detained persons was already released at the follow-up measurements. The dropout, therefore, was not produced by non-response, but rather reflects the Dutch prison system with its relatively short sentence lengths. However, sentence length corresponds with offense severity, which could have led to an overproportioned representation of individuals with more severe index offenses.

Third, mental health problems were only measured by a self-report instrument. The BSI total score reflects a number of different but rather mild symptoms that have been showing to be more resistant to changes. The instrument does not measure psychiatric diagnoses. Therefore, an exciting way for future research would be to assess distinct trajectories in symptoms of specific psychiatric disorders and their effect on recidivism.

## Conclusion

In this study, we identified different trajectories of mental health problems in a cohort of persons in pre-trial detention. The majority of participants displayed a low level of mental health problems remaining stable over time. By contrast, a smaller group of individuals reported high levels of mental distress that improved significantly during imprisonment. Group membership was associated with several sociodemographic and psychological characteristics indicating a high burden of pre-existing personal and mental health problems in the second group. These findings stress the need for correctional mental health services to improve detained persons' mental health. However, we could not find an association of membership in one of the two identified groups with criminal recidivism. An interesting avenue for future research would be to examine changes in specific mental health symptoms or disorders in relation to recidivism risk. Furthermore, research on the effectiveness of therapeutic interventions aiming at a positive effect on detained persons' mental health is needed.

## Data availability statement

The datasets presented in this article are not readily available but interested authors are welcome to request for collaboration from the principal investigators of the Prison Project. Requests to access the datasets should be directed to http://www.prisonproject.nl/eng/.

## Ethics statement

The studies involving human participants were reviewed and approved by Ethical Committee for Legal and Criminological Research of the VU University Amsterdam. The patients/participants provided their written informed consent to participate in this study.

## Author contributions

MW, SB, and AD designed the study. AD and PN collected the data. SB analyzed the data with the involvement of LG, MW, and AD. MW took the lead in writing the manuscript in close collaboration with SB, LG, PN, and AD. All authors provided critical feedback and helped shape the design, analyses, interpretation, and manuscript.

## Funding

Data were collected as part of the Prison Project, which was financially supported by Leiden University, Netherlands Institute for the Study of Crime and Law Enforcement, the Netherlands Organization for Scientific Research (VICI grant 453-08-005), and Utrecht University.

## Conflict of interest

The authors declare that the research was conducted in the absence of any commercial or financial relationships that could be construed as a potential conflict of interest.

## Publisher's note

All claims expressed in this article are solely those of the authors and do not necessarily represent those of their affiliated organizations, or those of the publisher, the editors and the reviewers. Any product that may be evaluated in this article, or claim that may be made by its manufacturer, is not guaranteed or endorsed by the publisher.

## Supplementary material

Supplementary Material 1 Method of systematic literature research on factors associated with changes in mental health status of detained persons.

Supplementary Table 1 Description of trajectories: maximum likelihood estimates (main analysis; censored normal model; *N* = 1,635).

Supplementary Table 2 Description of trajectories: maximum likelihood estimates (sensitivity analysis; censored normal model; *N* = 223).

Supplementary Table 3 Predictors for membership in trajectory group 2 (sensitivity analysis; *N* = 223).

Supplementary Figure 1 Flow diagram of systematic literature search on factors associated with mental health changes during imprisonment. Adapted from: Page MJ, McKenzie JE, Bossuyt PM, Boutron I, Hoffmann TC, Mulrow CD, et al. The PRISMA 2020 statement: an updated guideline for reporting systematic reviews. *BMJ*. (2021) 372:n71. doi: 10.1136/bmj.n71.

Supplementary Figure 2 GBTM revealing two distinct groups of mental health trajectories (sensitivity analysis; *N* = 223).
