# Supplementary material for: Longitudinal trajectories of mental health problems and their association with reoffending in a Dutch pre-trial prison cohort
Source: Front Psychiatry. 2022 Sep 9;13:976832. doi: 10.3389/fpsyt.2022.976832 (PMC9504669; doi:10.3389/fpsyt.2022.976832)
Supplement: Supplementary Material 1 — Method of systematic literature research on factors associated with changes in mental health status of detained persons. [file Data_Sheet_1.DOCX]

Supplementary Material

# Supplementary Material 1. Method of Systematic Literature Research on Factors Associated With Changes in Mental Health Status of Detained Persons.

To identify factors known to predict mental health changes in detained persons, a systematic review in the PsycInfo Database (EBSCO interface; 2002 onwards) was conducted. The following search terms were applied: “( ( TI (prison* or incarcerat*) AND TI ( mental health or mental illness or mental disorder or psychiatric illness ) AND TI change ) ) OR ( ( AB (prison* or incarcerat*) AND AB ( mental health or mental illness or mental disorder or psychiatric illness ) AND AB change ) )”.

# Supplementary Table 1. Description of Trajectories: Maximum Likelihood Estimates (Main Analysis; Censored Normal Model; *N* = 1,635).

| Group | Parameter | Estimate | Standard Error | T for H0: Parameter = 0 | Prob > \|T\| |
| --- | --- | --- | --- | --- | --- |
| 1 | Intercept | 0.33058 | .08 | 4.073 | < .001 |
|  | Linear | 0.22095 | .10 | 1.088 | .277 |
|  | Quadratic | -0.03576 | .03 | -1.285 | .199 |
|  |  |  |  |  |  |
| 2 | Intercept | 3.11355 | .17 | 18.244 | < .001 |
|  | Linear | -1.54174 | .21 | -7.476 | < .001 |
|  | Quadratic | 0.28886 | .05 | 5.257 | < .001 |
|  |  |  |  |  |  |
|  | Sigma | 0.46355 | .01 | 61.992 | < .001 |

# Supplementary Table 2. Description of Trajectories: Maximum Likelihood Estimates (Sensitivity Analysis; Censored Normal Model; *N* = 223).

| Group | Parameter | Estimate | Standard Error | T for H0: Parameter = 0 | Prob > \|T\| |
| --- | --- | --- | --- | --- | --- |
| 1 | Intercept | 0.37 | 0.19 | 1.968 | .050 |
|  | Linear | 0.15 | 0.22 | 0.674 | .500 |
|  | Quadratic | -0.05 | 0.05 | -0.971 | .332 |
|  |  |  |  |  |  |
| 2 | Intercept | 2.84 | 0.37 | 7.601 | < .001 |
|  | Linear | -1.20 | 0.41 | -2.916 | .004 |
|  | Quadratic | 0.21 | 0.10 | 2.051 | .041 |
|  |  |  |  |  |  |
|  | Sigma | 0.51 | 0.02 | 31.467 | < .001 |

# Supplementary Table 3. Predictors for Membership in Trajectory Group 2 (Sensitivity Analysis; *N* = 223).

| Variable | Coefficient | Standard Error | T | p |
| --- | --- | --- | --- | --- |
| Age | 0.00 | 0.02 | 0.22 | .827 |
| Education (medium/high) | 0.05 | 0.46 | 0.10 | .918 |
| Work (yes) | -0.15 | 0.46 | -0.33 | .742 |
| Debts (yes) | 0.46 | 0.48 | 0.97 | .335 |
| Violent offending (yes) | 0.14 | 0.46 | 0.30 | .761 |
| D1 Substance use problems (yes) | 1.08 | 0.50 | 2.15 | .032 |
| D1 Any psychiatric disorder (yes) | 1.47 | 0.50 | 2.91 | .004 |
| Psychiatric care in detention (yes) | 0.37 | 0.60 | 0.62 | .536 |
| D1 Subjective experience of detention | 0.95 | 0.29 | 3.30 | .001 |


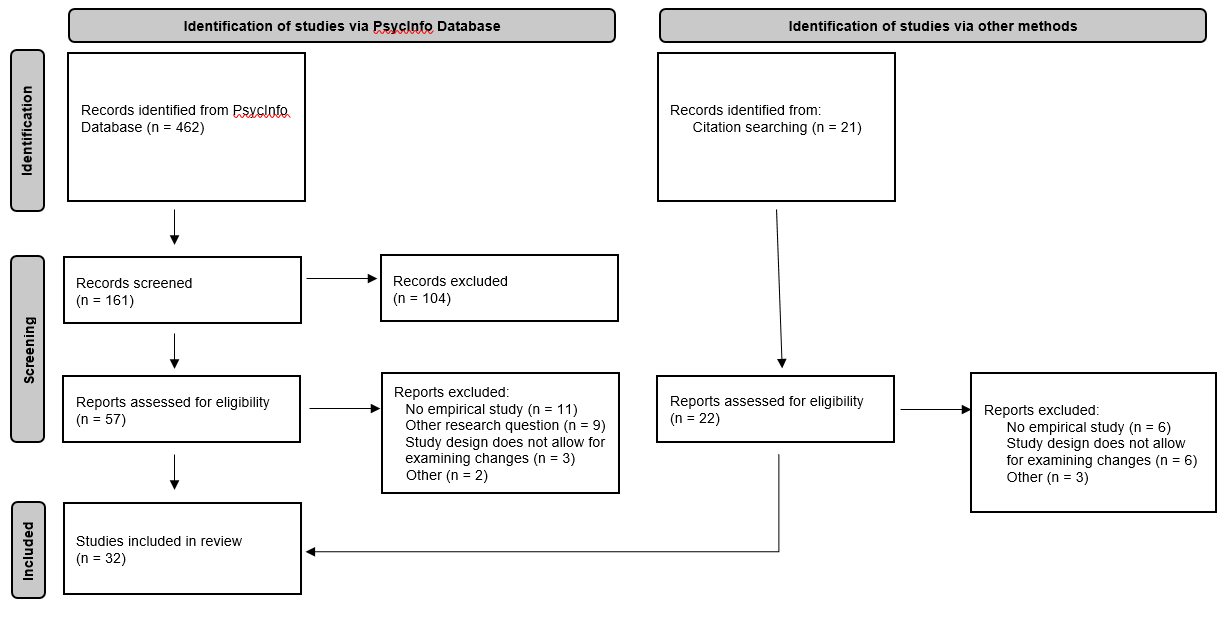


# Supplementary Figure 1. Flow Diagram of Systematic Literature Search on Factors Associated with Mental Health Changes During Imprisonment. Adapted from: Page MJ, McKenzie JE, Bossuyt PM, Boutron I, Hoffmann TC, Mulrow CD, et al. The PRISMA 2020 statement: an updated guideline for reporting systematic reviews. BMJ 2021;372:n71. doi: 10.1136/bmj.n71.


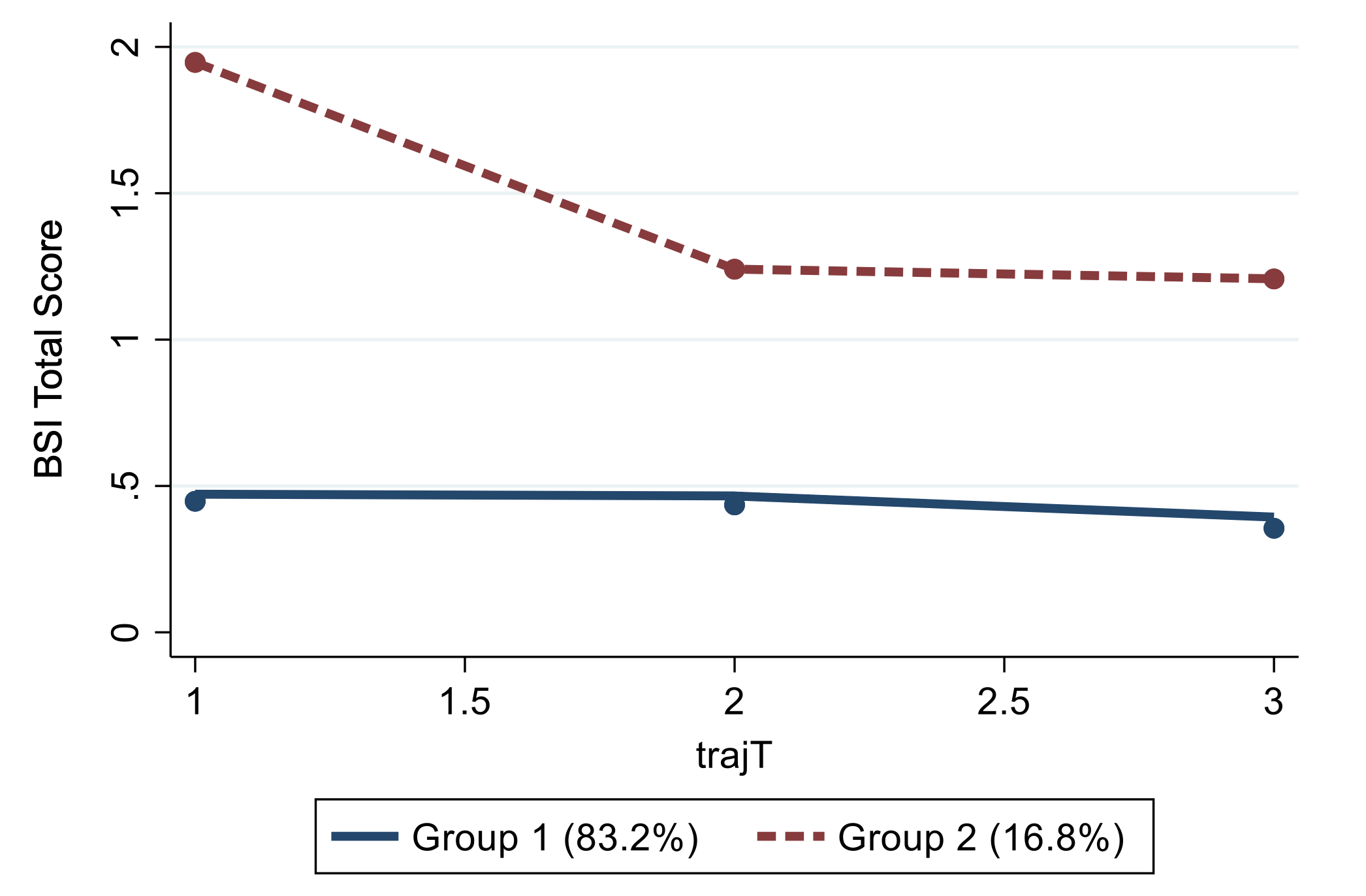


# Supplementary Figure 2. GBTM revealing two distinct groups of mental health trajectories (Sensitivity Analysis; *N* = 223).
